# Applying Translational Science Approaches to Protect Workers Exposed to Nanomaterials

**DOI:** 10.3389/fpubh.2022.816578

**Published:** 2022-06-10

**Authors:** Paul A. Schulte, Rebecca J. Guerin, Thomas R. Cunningham, Laura Hodson, Vladimir Murashov, Borsika Adrienn Rabin

**Affiliations:** ^1^Advanced Technologies and Laboratories (ATL) International, Inc., Gaithersburg, MD, United States; ^2^National Institute for Occupational Safety and Health, Centers for Disease Control and Prevention, Cincinnati, OH, United States; ^3^National Institute for Occupational Safety and Health, Centers for Disease Control and Prevention, Washington, DC, United States; ^4^University of California, San Diego, San Diego, CA, United States

**Keywords:** dissemination, implementation, nanotechnology, research, impact

## Abstract

Like nanotechnology, translational science is a relatively new and transdisciplinary field. Translational science in occupational safety and health (OSH) focuses on the process of taking scientific knowledge for the protection of workers from the lab to the field (i.e., the worksite/workplace) and back again. Translational science has been conceptualized as having multiple phases of research along a continuum, beyond scientific discovery (T_0_), to efficacy (T_1_), to effectiveness (T_2_), to dissemination and implementation (D&I) (T_3_), to outcomes and effectiveness research in populations (T_4_). The translational research process applied to occupational exposure to nanomaterials might involve similar phases. This builds on basic and efficacy research (T_0_ and T_1_) in the areas of toxicology, epidemiology, industrial hygiene, medicine and engineering. In T_2_, research and evidence syntheses and guidance and recommendations to protect workers may be developed and assessed for effectiveness. In T_3_, emphasis is needed on D&I research to explore the multilevel barriers and facilitators to nanotechnology risk control information/research adoption, use, and sustainment in workplaces. D&I research for nanomaterial exposures should focus on assessing sources of information and evidence to be disseminated /implemented in complex and dynamic workplaces, how policy-makers and employers use this information in diverse contexts to protect workers, how stakeholders inform these critical processes, and what barriers impede and facilitate multilevel decision-making for the protection of nanotechnology workers. The T_4_ phase focuses on how effective efforts to prevent occupational exposure to nanomaterials along the research continuum contribute to large-scale impact in terms of worker safety, health and wellbeing (T_4_). Stakeholder input and engagement is critical to all stages of the translational research process. This paper will provide: (1) an illustration of the translational research continuum for occupational exposure to nanomaterials; and (2) a discussion of opportunities for applying D&I science to increase the effectiveness, uptake, integration, sustainability, and impact of interventions to protect the health and wellbeing of workers in the nanotechnology field.

## Introduction

**Figure 1:**
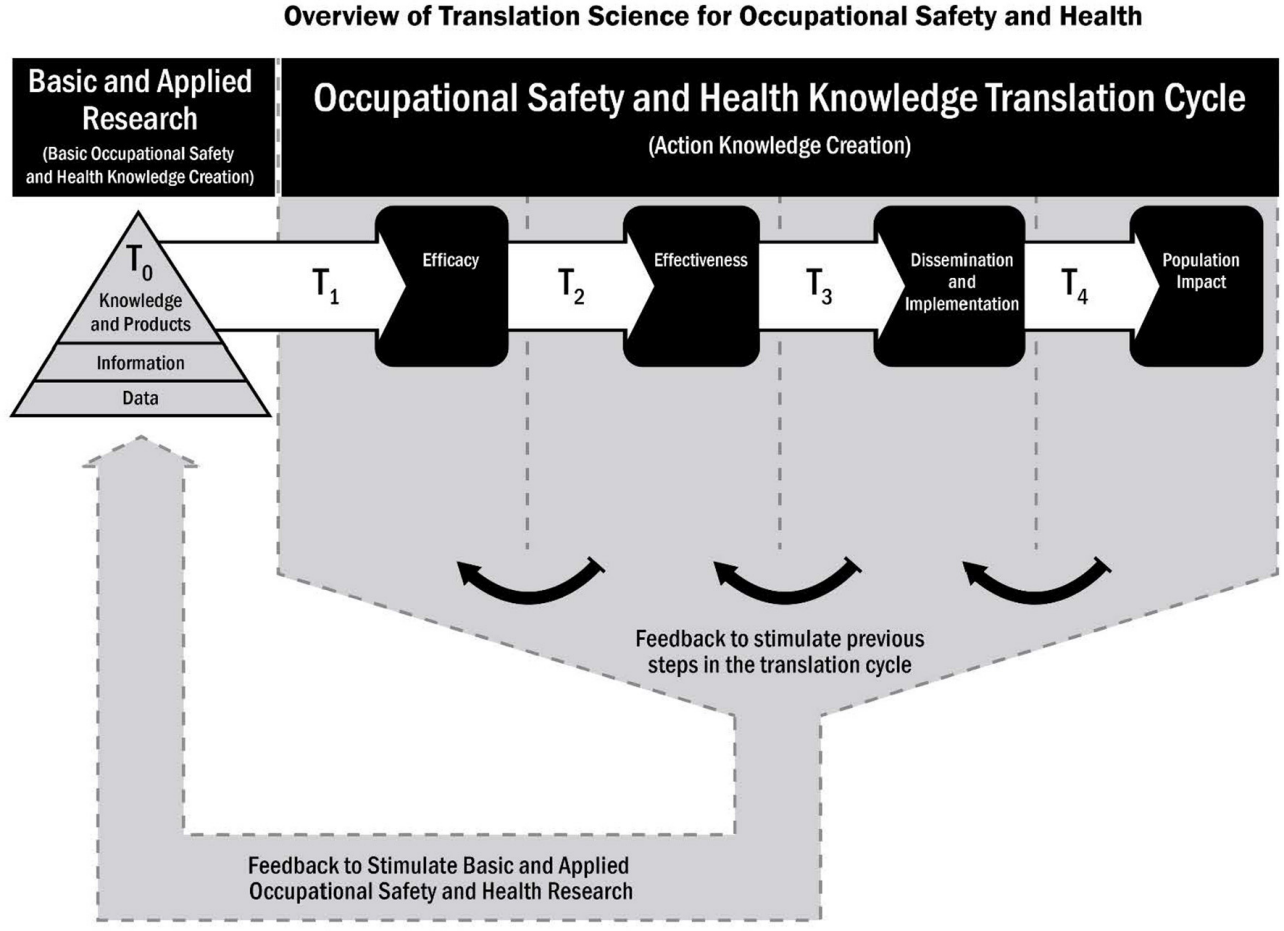
Overview of translational science for occupational safety and health [adapted from Schulte et al. (11)].

Nanotechnology and the development of nanomaterials have yielded a large number of commercial products and exposures to workers (1–3). The diffusion of nanotechnology into commerce in the early 2000's raised concern about potential hazards to workers (4–6). The small particle size, novel characteristics of nanoparticles and hypotheses about interaction with biological molecules and structures prompted public health authorities to consider whether workers could be at risk of adverse effects from exposure to engineered nanomaterials (ENM). To prevent such effects, if they were to occur, occupational public health authorities promoted and conducted research and issued guidance for employers and workers on risk management (6–8). The overarching questions are identifying the extent to which guidance was used, and whether the guidance made a difference in the health and safety of workers. Unfortunately, these and related questions have not been the focus of much research. In fact, such research has rarely, with notable exceptions, been considered or implemented in the occupational health field, in general, let alone in the area of exposure of workers to ENM (9–11). To address this shortfall there has been an effort in recent years to promote research on the “downstream” part of a continuum beyond basic research to impact (Figure 1) (11). This research, which includes dissemination and implementation (D&I) research, can fall under the umbrella of “translational science.” Traditionally, translational science has focused largely on barriers to intervention development at the efficacy and effectiveness stages (T_1_ and T_2_). Once an evidence base is established, D&I science has focused on barriers to the adoption, use, and sustainment (T_3_ and T_4_) of evidence-based interventions (12). Table 1 illustrates key characteristics of dissemination and implementation research and hypothetical examples for the occupational safety and health field. Figure 1 depicts the translational science cycle, conceptualized as crossing all translational or “T” phases of the research continuum, from scientific discovery (T_0_) to efficacy (T_1_), effectiveness (T_2_), dissemination and implementation (T_3_), and the outcomes and effectiveness and impact of research in populations (T_4_). OSH interventions (such as those represented in Table 2) may be situated anywhere along the research continuum, and, in reality, do not necessarily pass through all the T stages. In the context of OSH research (and beyond), there is a dearth of research conducted in the T_3_ and T_4_ stages of the research continuum (14).

**Table 1:** Ideas for advancing dissemination and implementation science in occupational safety and health.

**Key characteristics to consider for dissemination and implementation (D&I) research for occupational safety and health (OSH):**
• Understand how effective OSH interventions work, particularly multi-level or multi-component interventions, to inform how those interventions can optimally be delivered when implemented in various workplace settings. • Understand the relevance of OSH interventions, where applicable, to meet the needs of underserved populations and/or low-resource settings. • Incorporate theories, models, and/or frameworks appropriate for D&I to inform study hypotheses, measures, and outcomes. • Consider extant literature on barriers to and facilitators of the dissemination and implementation of practices to improve worker's OSH. • Consider and characterize the multi-level context and environment in which the proposed research will be conducted. • Consider the use of qualitative and/or mixed methods approaches. • Focus on issues of resources expended, program costs, cost-effectiveness, or other economic outcomes related to dissemination and/or implementation. • Incorporate stakeholder-relevant outcomes (i.e., outcomes relevant to workers, employers, insurers, and/or policymakers).
**Hypothetical examples of relevant research topics may include but are not limited to:**
• Studies of the implementation of multiple evidence-based practices within businesses and sectors to meet the needs of employers and workers. • Studies of the business adaptation of evidence-based OSH practices in the context of implementation. • Longitudinal and follow-up studies on the factors that contribute to the sustainability of evidence-based interventions in business sectors that lead to the reduction in work-related morbidity and mortality. • Studies of the relationship of context and local capacity of business and sector settings to adoption, implementation, and sustainability of evidence-based practices. • Studies of influences on the creation, packaging, transmission, and reception of evidence for effective OSH interventions. • Studies of strategies to impact organizational structure, safety climate, safety culture, and processes to enable D&I of OSH information and effective OSH interventions. • Studies that focus on the testing of relevant D&I theories, models, and frameworks. • Studies of policies and other contextual factors that influence the success of dissemination or implementation efforts.

*Adapted from NIH (13)*.

**Table 2:** Examples of interventions to address worker exposure to ENMs.

**Intervention**	**Example**
Program	OECD Testing Programmers of Manufactured Nanomaterials
Practices	Occupational Exposure Limits
Principle	Consider ENM hazardous until proven otherwise
Procedures	Nanomaterial Exposure Assessment Technique
Products	Direct reading instrumentation
Policies	ISO/TC 229 Nanotechnologies; WHO guidance on protecting workers from potential risks of manufactured nanomaterials.

While closely connected, the primary focus of translational science is *not* to do translation or its synonymous activity, research-to-practice (R2P) [an approach to collaborations with partners on the adoption, use, and adaptation of knowledge, interventions and technologies (15)]. Rather, it is the study of how these activities and processes (i.e., uptake, implementation, sustained use) work, what their multilevel barriers and facilitators are, and what intermediate and ultimate impacts they have on diverse outcomes. It should be noted that the processes shown in Figure 1 are not linear but represent a dynamic and iterative interplay across stages and involve many factors that interact and provide feedback to previous and consecutive stages. The challenge is to study the main causal factors while accounting for and acknowledging the complex context within which they exist.

Ultimately, there is a need to investigate many casual pathways that influence worker safety and health outcomes. Sorensen et al. (16) developed a conceptual model “based on the premise that addressing multiple pathways in an integrated manner with a focus on the conditions of work will contribute to greater impairment in worker and enterprise outcomes than addressing each pathway separately” (16). This integrated approached can guide research and intervention designs and may be a framework for T_1_ and T_2_ efforts.

In this paper we examine how research and guidance on ENMs are situated within the translational science continuum, and identify gaps related to, and opportunities for, increasing research efforts particularly at the later stages (T_3_ and T_4_) of that continuum. To that end, the paper will provide: (1) an illustration of the translational research continuum for occupational exposure to ENMs; and (2) a discussion of opportunities for expanding efforts in D&I science to increase the effectiveness, integration and impact of interventions to protect the health and wellbeing of workers in the nanotechnology field.

For illustrative purposes, we present three examples of OSH interventions— broadly defined as programs, practices, policies, recommendations, and guidelines (17)—that can be considered relevant for occupational exposure to ENMs and could give rise to translational research questions: (1) National Institute for Occupational Safety and Health (NIOSH) recommended exposure limits for titanium dioxide (TiO_2_) and carbon nanotubes and nanofibers, (CNTs/CNFs) (18, 19); (2) World Health Organization (WHO) guidelines for protecting workers (20); and (3) The International Risk Governance Council (IRGC) guidance on nanotechnology risk governance (21, 22). These examples were selected because they are widely known by investigators and practitioners in the field of nanomaterial workers' health and illustrate how translational science can be applied to different situations.

### What Is the Evidence and What Are Interventions for ENM?

Critical in translational science is understanding how evidence is defined. When is it decided that the science or assembled information is sound enough to transmit to end users? (23). Too much waiting for interventions to meet specific evidence standards may contribute to translational lag while also leading to the generation of interventions that cannot be replicated in real-world settings such as worksites (24, 25). Applying a translational science lens to the development and implementation of interventions related to ENMs might change our definition of evidence and what activities can be undertaken depending on the stage on the translational science continuum. While the evidence for basic science is well-established and is based on more traditional scientific experimentation randomized control trials (RCT), evidence at later stages of the continuum may vary and can be based on the synthesis of information and peer review of guidance or a critical review of the literature based on results from diverse study designs (e.g., cross-sectional, case control, or longitudinal studies). When establishing evidence for later stage translational work, traditional RCTs can become problematic. For example, the use of RCTs in occupational settings, arguably, could be seen as unethical if an intervention judged to be efficacious and safety or health-enhancing is withheld from some segment of the workforce exposed to a potential hazard (26, 27).

It is also important to identify what an intervention is with regard to ENMs. The interventions to protect workers from adverse effects may vary substantially. Generally, in translational science, interventions can be described in broad terms as one of the “7 P's”: programs, practices, principles, procedures, products, pills and policies (28). In the ENM arena most of these, except pills, would be considered as interventions to address occupational hazards (Table 2). Table 2 was developed to help the nanotechnology community understand that “intervention” can have a wide range of meanings. These interventions can fit at all stages of Figure 1. The target audiences for translational science are the organizations and decision-makers who develop, disseminate, or implement interventions. These audiences include government authorities, decision-makers, employers, trade associations, and unions. Ultimately the interventions will be implemented to protect workers but the pathway generally will be through employers.

## Translational Science

It is useful to envision the continuum of translational science from beyond basic research through the impact of that research on populations. Translational science has been conceptualized as having multiple phases of research beyond scientific discovery (T_0_), to efficacy research (T_1_), to effectiveness research (T_2_), to dissemination and implementation research (D&I) (T_3_), to outcomes and effectiveness research in populations (T_4_) (12, 29–35). From an occupational safety and health perspective, for translational research findings to make a difference in the safety and health of workers it is necessary to determine the best ways to direct basic research findings and guidance information to employers, workers and authorities promotes the uptake of that information, and determines whether it made a positive impact.

The T_0_ stage is focused on scientific discovery driven by basic science. While there are important feedback loops between the T_0_ and later T_1_ through T_4_ stages, this paper will focus on T_1_ to T_4_ that is, how well the products of basic research can be utilized to affect and protect ENM workers and, specifically, how to study the stages leading to such protection. To better view the stages between T_1_ and T_4_ this paper reviews various examples of the continuum applied to hazard information and interventions related to ENMs. In the next sections and in Tables 3–**5** we present three examples.

**Table 3:** Utilization of translational science pertaining to NIOSH recommended exposure limits for TiO_2_ and CNT/CNF.

**Example 1: Recommended exposure limits (CNT/CNF & TiO_2_)**		
T_0_	Basic science	Ultrafine and fiber toxicity Specific studies of ENMs Quantitative risk assessment
T_1_	Efficacy research	Sensitivity analysis of risk assessment Historical basis for OELs
T_2_	Effectiveness research	Citation analysis/downloads Cross-sectional study (CNT)
T_3_	Dissemination & implementation research	No examples Hypothetical questions
T_4_	Population impact	Use of intermediate indicators [e.g., (36)] Longitudinal studies

*NIOSH (18, 19)*.

Translational science is a field that merits support because it is a crucial that stakeholders receive and use recommendations and interventions as a means to responsible development of a technology, corporate responsibility, and worker and population wellbeing (37–42). The successful commercialization of nanotechnology depends on societal acceptance of it. Societal acceptance will be influenced by, among other things, whether workers are protected from nanotechnology hazards. When it is apparent that workers are at high risk of adverse health effects and not protected, the societal response toward acceptance of the technology is likely to be negative (37). Therefore, it is incumbent on employers and authorities to support the development of translational science as a means to support responsible development of nanotechnology. Supporting translational science will result in broad utilization of guidance and increase involvement of stakeholders in that process (37, 43–47). For example, Brownson (47), building on work of Curry (48) and Anderson et al. (49), described a push-pull model for strategic public health science that could be adopted to strategic transitional science for occupational safety and health: “This model posits that for science to effect practice there must be a combination of push (a basis in science and technology) and the pull (a market demand from practitioners), and the capacity (delivery ability of public health systems).” The adaption would be that practitioners would be considered as employers, workers, unions, trade associations, and other decision-makers that affect worker protection.

### Example 1: NIOSH Recommended Exposure Limits for TiO_2_ and CNT

#### T_0_: Basic Science - Discovery Data

Table 3 illustrates the translational science continuum for this example. Titanium dioxide (TiO_2_) and CNT/CNF are ENMs that were studied early on in the commercialization of nanotechnology [see (18, 19) for review] and found to have adverse health effects in laboratory animals. Clearly, there are many types of TiO_2_, and CNT/CNF and the toxicity information only pertained to specific chemical and physical types. The type of toxicological assessments that is most relevant to occupational safety and health are rodent inhalation studies. TiO_2_ and other poorly soluble particles of low toxicity (PSLT) of fine and ultrafine sizes were found to show consistent dose-response relationships for adverse pulmonary inflammation and lung tumors when dose was expressed as particle surface area. Specifically, the chronic animal inhalation study of TiO_2_ (50) demonstrated the development of lung tumors (bronchioloalveolar adenomas) in response to exposure to a relatively large dose of 250 mg/m^3^.

Subsequently, Henrich et al. (51) showed a statistically significant lung cancer excess in rats exposed to ultrafine TiO_2_ at a concentration of 10 mg/m^3^.

Studies in rats and mice have shown that CNTs and CNFs can pose a respiratory hazard due to pulmonary inflammation and rapidly developing, persistent fibrosis (19, 52). Occupational exposure to CNTs and CNFs has been associated with biomarkers of fibrosis, inflammation, oxidative stress, and cardiovascular responses in workers (53).

#### T_1_: Efficacy Research

The translational science T_1_ phase refers to the development of an intervention and studies of its efficacy, which is a term to describe whether an intervention can work in an optimal and highly controlled laboratory setting. For controlling exposures, occupational exposure limits (OELs) [for example, NIOSH Recommended Exposure Limits (RELs)] are well-established interventions (7, 18, 19). The focus of this example are the NIOSH RELs for TiO_2_ and CNTs/CNFs. The RELs are generally based on quantitative risk assessments of appropriate toxicity data sets. In the realm of ENMs T_1_ can be thought of as whether a risk assessment is robust. For TiO_2_, animal data were analyzed in a dose-responsive quantitative risk assessment and two categories of TiO_2_ were assessed: fine (>100 nm) and ultrafine (≤100 nm), and respective RELs of 2.4 and 0.3 mg/m^3^, as a time-weighted average concentration for up to 10 h per day during a 40 h work week were determined. These RELs corresponded to lifetime risk estimates associated with <1/1,000 excess risk for lung cancer. To assess the efficacy of this risk determination a model averaging approach was utilized. This approach uses all the information from many exposure-response models and weights them by the Akaike information criteria for model fit and constructs an average dose-response model (54, 55). CNT animal studies indicated the CNT exposure may result in localized and systematic inflammation, cytotoxicity, pulmonary, interstitial fibrosis, mutagenesis, and the potential for lung cancer (19). NIOSH performed a quantitative risk assessment using dose-response data of adverse lung effects in rats following subchronic inhalation of CNTs and CNFs, and also evaluated rodent studies of lung effects by other routes of exposure. The NIOSH REL of 1 μg/m^3^ for CNTs/CNFs (as a respirable mass 8-h TWA concentration) was set at the limit of quantification (LOQ) of the analytical method for element carbon (NIOSH method 5040).

In addition to the RELs for TiO_2_ and CNT/CNFs, NIOSH provided guidance for hazard and risk management in the NIOSH Current Intelligence Bulletins for TiO_2_ and CNT/CNF that could be considered for expository purposes as part of the intervention (18, 19). The T_1_ efficacy research for this intervention included peer, stakeholder, and public reviews. In general, these reviews supported and validated the recommendations in the Current Intelligence Bulletins.

#### T_2_: Effectiveness Research

Studies conducted under the T_2_ stage of the translational continuum are focused on assessing the effectiveness of an intervention. This refers to whether the intervention “works” in the real world outside the laboratory where the conditions are less controlled. Can it protect workers and prevent disease from exposure to TiO_2_ and CNT/CNF? And can results from these studies be generalized to other ENM workers/settings?

While not specifically designed as a T_2_ study, a cross-sectional study showed that in 12 facilities handling CNT/CNFs that approximately 93% of averaged samples collected at the respirable fraction for EC mass were below the NIOSH REL of 1 μ/m^3^ (56). This indicates that the REL could be met in practice. No other studies were identified to assess the effectiveness of the NIOSH RELs.

#### T_3_: Dissemination and Implementation Research

Dissemination and implementation (D&I) science is defined as the study of methods and strategies for bridging the gap between public health research and practice (13). D&I scientists use a number of empirically tested theories, models and frameworks (TMFs) to plan, evaluate, or understand barriers and facilitators to D&I processes (57–62). Some commonly used TMFs among D&I researchers (63) include the Consolidated Framework for Implementation Research (CFIR) (57), the RE-AIM (Reach, Effectiveness, Adoption, Implementation, Maintenance) framework and its contextually expanded version the Practical Robust Implementation and Sustainability model (PRISM) (61, 64, 65), the EPIS (Exploration, Planning, Implementation, Sustainment) framework (66, 67), the Diffusion of Innovation theory (68), and the Theoretical Domains Framework (TDF) (69). However, more than 150 D&I TMFs have been identified (70).

In regard to nanotechnology and worker's health dissemination is the active and targeted distribution of information and intervention materials to a specific business audience. Critical to this is the involvement/engagement with stakeholders early on in the research process so the interventions and strategies for distribution are feasible, relevant, and equitable. Dissemination research is defined as the scientific study of these phenomena for the purpose of understanding how best to spread knowledge required to adopt or deploy an intervention. Implementation of the REL is the critical intermediate step in the continuum from research to prevention. Implementation can be defined as the adoption and integration of evidence-based health intervention (in this case RELs) into business practices.

Implementation research is the study of this process (12). Important translational research questions include what prompts employers to consider the uptake of, and implementation into routine practice and use of such guidance, and what are the key barriers and facilitators to the use of those interventions across diverse workplace settings with different resources (14). D&I science inquiry addresses what works, for who, how, why, in what settings, and how it is sustained over time (34).

Implementation strategies that might be relevant for study include engaging trusted intermediaries and developing a business case.

In the T_3_ D&I research stage, there are many rigorous study designs that have been used (28, 71). “These include both experimental (e.g., randomized controlled trial, cluster-randomized controlled trial, pragmatic trial, stepped wedge trial, dynamic wait-listed control trial) and quasi- experimental (e.g., nonequivalent groups, pre-/post-, regression discontinuity, interrupted time series), non-experimental or observational (including designs from epidemiology) designs as well as qualitative (e.g., focus groups, semi-structured interviews), mixed-methods (i.e., the collection and integration of qualitative and quantitative data), and system science (e.g., system dynamics, agent-based modeling) approaches” (71). Except for a few qualitative studies, rarely, have any of these study designs been applied to ENM interventions (36).

It appears that there are no published examples of T_3_ D&I studies related to ENM safety and health so we provide a hypothetical example of questions that could be investigated. Numerous law firms, trade associations, and labor groups served to amplify the REL information published by NIOSH. Translational research in the T_3_ stage could address what were the best strategies to get the REL information (i.e., intervention) to employers, what are the factors that hinder/facilitate adoption of REL information by employers, how REL information should be packaged and what additional information and resources are needed to facilitate broad dissemination, uptake, and initial implementation by organizations and what are key strategies and resources needed for the sustained use of REL information over time across diverse workplaces. It is critical at this stage and all stages of the research continuum, to consider questions around occupational health equity. These include: how diverse stakeholders including employers, employees labor groups, and professional organizations, will be engaged in the research process and the delivery and implementation of the intervention (i.e., REL information); and will the intervention be feasible for lower resource workplaces employing individuals with high vulnerability?

Measuring success in the T_3_ stage can take various forms. A possible indicator of success might be to assess the extent to which an intervention, in this case NIOSH guidance, was adopted by companies as the basis for risk management efforts for TiO_2_ exposure. To make this determination it would be necessary to survey employers on whether they based risk management decisions on NIOSH guidance. However, for such surveys it is difficult to get appropriate participation possibly because the information requested may be viewed as “business confidential”. It may be that tracking the uptake and use of specific guidance may be quite difficult and it might be better to attempt to track uptake of authoritative guidance in general.

Moreover, tracking use of guidance assumes a linear process that a certain authoritative report will influence an employer to take action when clearly employer decision-making is influenced by a large number of factors.

#### T_4_: Population Impact

Cross-sectional data shown under the T_2_ effectiveness stage indirectly demonstrates the population impact of REL information within the population of ENM workers and employers. Although due to the limitations of the design it is challenging to establish a causal pathway between the intervention (REL information) and the observed outcomes of REL usage and exposure control. More data are needed on potential impact of the REL intervention from workplaces where exposure could occur. Also needed are data on the prevalence of control use. There are few studies of the use of controls for ENM interventions in general. Iavicoli et al. (36) found that controls were used substantially but their results were based on a small response rate. This may in part be due to concerns about confidentiality of business information. Ultimately, there is need for studies of the extent of disease in workers over time. Ideally, these would be longitudinal studies or two point in time surveillance assessments (72) and would include diverse process and impact outcomes.

### Example 2: WHO Guidance: Protecting From Potential Risks of Manufactured Nanomaterials

#### T_0_: Basic Science - Discovery Data

Table 4 illustrates the translational science continuum for this example. In addition to the type of toxicity data discussed earlier for TiO_2_ and CNT/CNFs, discovery data may be driven from stakeholders' concerns. For ENMs it was noted in 2007 that “Non-governmental organizations (NGOs) had been active in calling for worker protection in emerging nanotechnology industries (https://www.who.int/occupational_health/background_review_1.6.12.pdf; line 10).” In 2010 a European Trade Union Confederation recommended application of the precautionary principle because of potential hazards of ENMs. The focus of this example is the WHO guidance on protecting workers from potential risks of manufactured nanomaterials (20). Assessment of the health impacts of new technology is one of the activities of the Global Plan of Action on Worker's Health adopted in 2007. WHO was concerned that the increased production and use of ENM, meant that workers would be at the “…front line of exposures to these materials placing them at an increased of potential adverse health effects (20).” These guidelines were meant to prevent such effects.

**Table 4:** WHO Guidance for protecting workers.

**Example 2: WHO guidance for protecting workers**		
T_0_	Basic science	Historical and contemporary toxicity data Stakeholder requests
T_1_	Efficacy research	PECO analysis International expert assessment
T_2_	Effectiveness research	Historical evidence Cross-sectional and prospective studies
T_3_	Dissemination & implementation research	Implementation plan No D&I research
T_4_	Population impact	No population data Use of intermediaries

*WHO (20)*.

#### T_1_: Efficacy Research

The determination of efficacy of control guidance (i.e., the intervention) was based on years of practice controlling ultrafine particles (6–8). This was supported by evidence-based literature. Specifically, for efficacy of the guidance information created by the WHO workgroup, they used the PECO (Population/Situation Exposure-Comparison-Outcome) approach and the GRADE (Grading of Recommendations Assessment, Development and Evaluation) framework for the judgement of the quality of the evidence (20). The target group for the guidance was in phase 1: policy makers from low- and medium-income countries and in phase 2, an implementation guide for employers and workers. The policy was designed to fill critical knowledge gaps such as: (1) Can an algorithm be developed to classify engineered nanoparticles by degree of potential hazards? (2) Which characteristics of particles and which measurement techniques should be used for the assessment of exposure to engineered nanoparticles? (3) What is the exposure to engineered nanoparticles in the workplace? (4) What are the limits of engineered controls and personal protective equipment (PPE) with regard to engineered nanoparticles? (5) What occupational health surveillance should be recommended for workers potentially exposed to engineered nanoparticles? (6) Should exposure registries be established for various groups of workers potentially exposed to engineered nanoparticles? (7) Should engineered nanoparticles be treated as “new” substances and evaluated for safety hazards? The guidance was developed by an international group of experts with the assistance of the WHO Global Network of Collaborating Centres who conducted systematic review studies. The guidelines were peer reviewed by external reviewers.

#### T_2_: Effectiveness Research

The effectiveness of the guidance can be assessed against past experience and is similar to the efficacy determination. As noted, one way to assess the effectiveness is through citation analysis. Translation research approaches could be used to determine the extent to which exposure data have been collected in locations where the guidance has been implemented. What was found? No studies on this aspect were identified.

#### T_3_: Dissemination and Implementation Research

The foundation of the D&I stage is the causal evidence that underlies the intervention and the organizational change envisioned. In the WHO guidelines the intervention is recommendations for the safe use of nanomaterials in the workplace.

The WHO Guideline Development Group (GDG) prepared an implementation plan, which included the following activities:

1. Translating to other languages;
2. Integrating the recommendations of the WHO guidelines into training courses on nanosafety (e.g., UNITAR);
3. Providing and disseminating information:
  - web communication;
  - conference presentations;
4. Providing information on the E.U. and U.S. regulations for nanomaterials in the workplace;
5. Developing Safety Data Sheets for nanomaterials in collaboration with the WHO/ILO International Chemical Safety Cards program and UN GHS: nanoscale titanium dioxide (TiO_2_) ICSC#1782 and nanoscale zinc oxide (ZnO);
6. Developing a corporate governance document in collaboration with OECD;
7. Developing a list of practical tools for guideline implementation;
8. Assessing the use of nanomaterials in countries;
9. Providing real-world examples on the use of hazard information and proposed OELs in countries and in workplaces.

The GDG started working on the first five activities but did not complete this plan due to competing demands and priorities. Various D&I research studies could examine aspects of the adoption, implementation and sustainment of this plan, however, no studies of the barriers or facilitators in the plan were conducted. This is what would be needed for T_3_ stage.

#### T_4_: Population Impact

There are no data to describe the population impact of the WHO report on intermediate indicators such as the use of recommended risk management practices or on ultimate indicators of morbidity or mortality.

### Example 3: The International Risk Governance Council (IRGC) White Paper on Nanotechnology Risk

Table 5 illustrates the translational science continuum for this example. The International Risk Governance Council commissioned Renn and Roco to develop a white paper [distilled into a journal article (21)] on a risk governance framework for emerging nanotechnology.

**Table 5:** International Risk Governance Council guidance.

**Example 3: International Risk Governance Council guidance**		
T_0_	Basic science	Historic and contemporary toxicity data Explosiveness data Stakeholder requests
T_1_	Efficacy research	Scoping review on deficit in guidance
T_2_	Effectiveness research	Synthesis of evidence for risk governance Applicability of guidelines
T_3_	Dissemination & implementation research	No studies Possible research: extent employers adopted guidance/best means of dissemination and implementation strategies
T_4_	Population Impact	No studies

*IRGC (22)*.

#### T_0_: Basic Science - Discovery Data

The many facets of emerging nanotechnology coupled with many unknowns and extensive societal concerns in the early 2000's indicated that a comprehensive governance guidance was needed to protect citizens in general and workers in particular who might be at risk. The basic concern was that ENMs may potentially have toxic effects on people exposed to them. The purpose of the IRGC white paper on nanotechnology risk governance was to present decision makers with a systematic and integrated approach to analyzing and managing anticipated risks from exposure to ENMs (22). While the white paper was expected to have interest to governments, industry, academia, and NGOs, the prime focus was on governments' responsibility for developing and implementing policies.

There was also concern based on the history of dust explosions that nanoparticles could also be explosive. The white paper stated that, “There seems to be no lower particle size limits below which dust explosions could not occur. It may be possible that the increased surface area of nanoparticles could also include the likelihood that they become self-charged and ignite (22).”

#### T_1_: Efficacy Research

T_1_ research also involves assessing the potential of the IRGC to recommend risk governance guidance (intervention) (22). An initial scoping workshop involving a broad range of stakeholders and experts from academia, government, industry, insurance, law, and NGOs, was aimed at identifying large governance deficits for nanotechnology. Surveys of experts like those at the scoping workshop were also conducted. These surveys and workshops indicated that industry was highly aware of the potential impact on levels of innovation that could be caused by inadequate risk governance of human health and the environment, and the perception of the public. The major focus identified in the survey responses was research on Environment, Health and Safety and, in particular, the development of guidelines for worker health and safety and the establishment of an international methodology and nomenclature (22).

#### T_2_: Effectiveness Research

While many people and groups participated in the development of the IRGC risk guidance this is not necessarily evidence that it is effective. It does support, to some extent, its application potential and acceptance. Translational research could include: synthesis of evidence for risk governance and risk management of fine dust and powders, in particular, as well as efforts to explore applicability of governance guidelines for specific situations. In general, evidence gathering on effectiveness and value of governance could also be accomplished by multi-stakeholder evaluation (73).

#### T_3_: Dissemination and Implementation Research

T_3_ D&I studies would focus on research on the dissemination and implementation of the IRGC risk guidance. There were no examples in the published literature of T_3_ studies for the IRGC risk guidance. In the T_3_ stage, assessing the barriers, facilitators, and best practices for the dissemination and implementation of the IRGC risk guidance intervention could be undertaken. For dissemination, one might investigate the extent to which employers and other decision-makers received the governance guidance, what were the best strategies for dissemination, and was there variation in who did and did not receive the guidance (i.e., equity perspective). For implementation, research could focus on how employers adopted, used, and sustained practices based on the IRGC risk guidance.

#### T_4_: Population Impact

Like the other examples, the ultimate impact of an intervention is prevention of or decrease in morbidity and mortality associated with the ENM hazard potential. However, the ultimate impact of prevention may take a long time to obtain and the pathway between guidance and prevention may be indirect and involve diverse factors that are difficult to identify. Translational research may need to focus on intermediate outcomes, chiefly utilization of risk assessment and management practices included in the guidance. There were no published examples of T_4_ studies for the IRGC risk guidance.

## Conclusion

If occupational safety and health research on ENMs is to lead to protection of workers exposed to ENMs, there is a need to assure that the full continuum from basic research to population impact is considered. Historically, there has not been sufficient focus on the stages that appear “downstream” from basic research. The types of evidence needed and factors and processes associated with these later stages of the continuum can substantially differ from those relevant in earlier stages. Furthermore, these later stages (i.e., understanding what will determine eventual dissemination, implementation, and broad impact of an intervention) can have an important impact on how earlier stages of research (i.e., basic science and efficacy) are done. This paper provides a framework for considering the application of translational science to protect workers exposed to ENMs. Three examples were provided to illustrate this application. The key issues in applying translational science to ENM exposures is the need to invest in such science and the ability to get the results of such research to decision-makers and employers. Translational science may also include explorations of the information uptake and use by employers and decision-makers and identification of barriers and facilitators to these functions.

Clearly, the uptake and use of translational science pertaining to ENMs occurs against a backdrop of societal rules, laws and norms with regard to worker protection. Nonetheless, the outputs of translational science can provide useful information that can lead to the protection of workers. This paper is a call for utilizing translational science on a regular basis. If that is to occur there is need for funding of a workforce with translational science skills. With limited funding for ENM research in general it may be necessary to shift funding for better balance along all phases of the research to impact continuum.

## Author Contributions

PS wrote the first draft. RG and BR made major revisions. TC, LH, and VM provided useful comments and example materials. All authors contributed to the article and approved the submitted version.

## Funding

This was developed as part of government work. PS initiated this paper as part of government work and subsequently under contract.

## Author Disclaimer

The findings and conclusions in this report are those of the authors and do not necessarily represent the official position of the National Institute for Occupational Safety and Health (NIOSH), Centers for Disease Control and Prevention (CDC). Mention of any company or product does not constitute endorsement by NIOSH, CDC.

## Conflict of Interest

PS was employed by the company Advanced Technologies and Laboratories (ATL) International, Inc., under contract with NIOSH. The remaining authors declare that the research was conducted in the absence of any commercial or financial relationships that could be construed as a potential conflict of interest.

## Publisher's Note

All claims expressed in this article are solely those of the authors and do not necessarily represent those of their affiliated organizations, or those of the publisher, the editors and the reviewers. Any product that may be evaluated in this article, or claim that may be made by its manufacturer, is not guaranteed or endorsed by the publisher.
